# Moyamoya disease in a 2-year-old patient from the middle east: a case report and literature review

**DOI:** 10.1097/MS9.0000000000001934

**Published:** 2024-03-15

**Authors:** Haneen Mahmoud Nimer Habes, Raneen B. Alshareef, Areen Amleh, Ali A.A. Doudin, Yousef Mahmoud Nimer Habes, Mohammed Abdulrazzak, Sharif Issa Basal

**Affiliations:** a Faculty of Medicine, Al-Quds University, Jerusalem; b Department of Neurosurgery, Intervention Neuroradiology, Al-Ahli Hospital, Hebron, Palestine; c Faculty of Medicine, University of Aleppo, Aleppo, Syria

**Keywords:** moyamoya disease, seizure, palestine, revascularization, case report

## Abstract

**Introduction and importance:**

Moyamoya disease (MMD) is a condition characterized by progressive narrowing of arteries in the brain and abnormal development of small collateral vessels. It is commonly found in East Asia but has never been reported in Palestine.

**Case presentation:**

A 2-year-old female, part of a twin born to non-consanguineous parents, presented with recurring seizures and developmental regression. The physical examination revealed signs of hypotonia, reflex abnormalities, and bilateral Babinski signs. Comprehensive laboratory tests and imaging investigations confirmed the diagnosis of MMD, marking this patient as the reported case in Palestine.

**Clinical discussion:**

The diagnostic criteria for this condition were revised in 2021 to focus on findings seen in angiography and magnetic resonance angiography (MRA) scans. MMD has not been curative so far, and the management is focused on preventing complications, sometimes with surgical revascularization, including its different approaches: direct, indirect, and a combination of both.

**Conclusion:**

This case highlights the importance of identifying MMD in regions where it is uncommon to be diagnosed. It emphasizes the need for diagnosis and appropriate intervention to reduce complications.

## Introduction and Importance

Highlights

- Moyamoya disease (MMD) is a progressive cerebral vasculopathy, potentially resulting in neurological consequences due to compromised blood flow to the brain.
- The highest prevalence of MMD is in East Asian countries, particularly Japan, China, and Korea; this case represents the first documented case of MMD in Palestine.
- Ischaemic events are the most common initial presentation, especially in children.

Moyamoya disease (MMD) is an infrequent cerebrovascular disorder characterized by persistent and advancing stenosis of the major intracranial arteries, leading to the subsequent development of microvascular networks or collaterals^1^. The term “Moyamoya” originates from Japanese and signifies a misty, blurry, or swollen appearance. This nomenclature is attributed to the angiographic presentation of MMD, which resembles a smoke-like puff^2^.

The frequency of MMD exhibits significant regional discrepancies, with the most elevated occurrences observed in East Asian nations, notably Japan, China, and Korea^3,4^. Epidemiological investigations conducted in Japan have revealed a prevalence rate ranging from 3.2 to 10.5 cases per 100 000 individuals, with a higher incidence among females^5,6^.

MMD is a progressive cerebral vasculopathy, potentially resulting in neurological consequences due to compromised blood flow to the brain. This most commonly manifests as an ischaemic stroke or transient ischaemic attack, which may be frequent. Also, it may present uncommonly with seizures as a result of ischaemic damage, which are more common in children than in adults, as in our case^7–9^.

The definitive diagnosis of this condition is based on conventional angiographic criteria that include stenosis of the terminal portion of the intracranial arteries, the development of collaterals, and bilateral lesions. Unfortunately, there is no definitive cure for MMD. Nevertheless, providing supportive care can alleviate the chances of complications. Regular imaging assessments play a crucial role in pinpointing individuals with a heightened susceptibility to future ischaemic and haemorrhagic events, guiding the decision for surgical revascularization when beneficial^10^. In Palestine, MMD has never been reported yet.

The current paper presents a case of a 2-year-old female Palestinian patient with a history of recurrent seizures and regression of previously acquired developmental milestones who was diagnosed through a cerebral catheterization.

### Case presentation

A 2-year-old female from Palestine, part of a twin for a non-consanguineous couple, presented to the hospital with a history of recurrent episodes of convulsions. The first episode occurred at 13 months old and began with a staring gaze, followed by right-sided tonic-clonic convulsions that progressed to involve her entire body; the convulsion lasted for 1 min, followed by a post-ictal period that persisted for 15 min. Initial responsiveness to Tegretol was noted, but the convulsions recurred at the age of 16 months, affecting her left side. Subsequent attacks at 20 and 24 months old displayed right-sided convulsions with mouth deviation to the right side and started to cause drooling from the mouth. Additionally, the mother noticed that her baby started to lose her previously acquired developmental milestones; she couldn’t walk or sit without support and had upper and lower limb weakness with limited social interaction. However, there was no history of head trauma, fever, vomiting, headache, or ear discharge. There was no significant family history of autoimmune disease, early stroke, or ischaemic heart disease.

The physical examination revealed central and peripheral hypotonia, exaggerated deep tendon reflexes bilaterally, and a positive Babinski sign bilaterally. Laboratory investigations included a complete metabolic panel, a complete blood count, arterial blood gases, blood cultures, a thrombophilia genetic study, and a hypercoagulability workup showed normal results. Moreover, anti-neutrophil cytoplasmic antibodies (C-ANCA), anticardiolipin IgG, antinuclear antibody (ANA), homocysteine, plasma amino acids, urine organic acids, and cerebrospinal fluid (CSF) amino acids all resulted in negative results. The echocardiogram, electrocardiogram (ECG), and chest X-ray were unremarkable. Electroencephalogram (EEG), lumbar puncture with CSF analysis, ophthalmic exam, and hearing examination showed no abnormalities.

**Figure 1:**
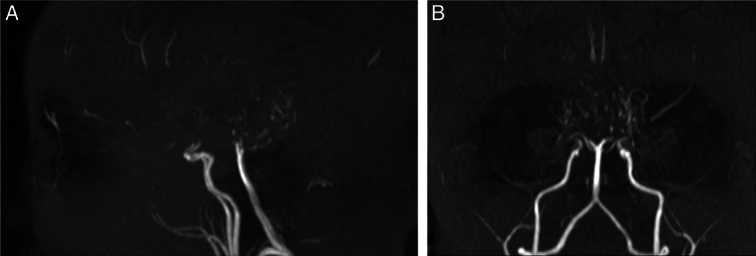
Time of flight: magnetic resonance angiography (TOF-MRA). (A) Lateral view. (B) Anteroposterior view. Complete occlusion of the terminal supraclinoid portions of both internal carotid arteries, with marked obliteration of the anterior, middle, and posterior cerebral arteries.

The brain computed tomography (CT) was nonspecific. Brain MRI, magnetic resonance angiography (MRA), and magnetic resonance venography (MRV) revealed complete occlusion of the terminal supraclinoid portions of both internal carotid arteries (ICA), marked obliteration of anterior, middle, and posterior cerebral arteries, and diffuse bilateral collateral formation. Meningeal hyperintensity and areas of cortical encephalomalacia suggested chronic ischaemic insults, consistent with MMD (Suzuki stage 4). MRA and MRV indicated multiple vascular narrowing, some with a beaded appearance (Fig. 1).

**Figure 2:**
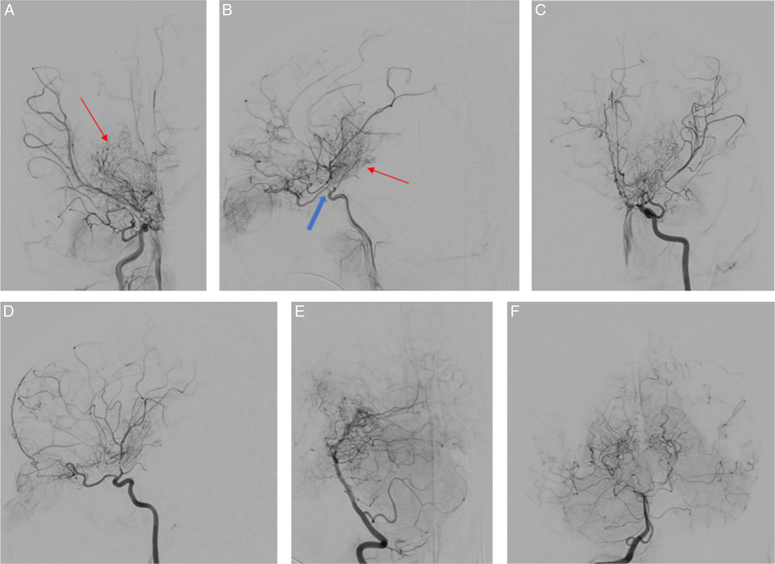
Cerebral digital subtraction angiography (DSA). (A) Right internal carotid arteries (ICA), AP view. (B) Right ICA, lateral view. Right ICA images show supraclinoid stenosis (blue arrow), with collaterals (puffy smoke) (red arrows), Moyamoya vessels from the ophthalmic artery, and no collaterals from external carotid arteries. (C) Left ICA, AP view. (D) Left ICA, lateral view. Left ICA: supraclinoid stenosis with collaterals (puffy smoke), Moyamoya vessels from the ophthalmic artery. (E) Right vertebral artery, lateral view. (F) Right vertebral artery, AP view. (E and F) are for the posterior circulation and show the right vertebral Moyamoya vessels.

Endovascular evaluation by cerebral catheterization was done for a definitive diagnosis and possible revascularization. Under general anaesthesia, cerebral catheterization revealed supraclinoid stenosis of the ICA with collaterals from the ipsilateral ophthalmic artery (puffy smoke). Moyamoya vessels were noted in the posterior circulation and bilateral vertebral arteries (Fig. 2). The patient was discharged on carbamazepin and Aspirin for outpatient follow-up. Revascularization surgery was planned.

## Discussion

East Asian nations, especially Korea and Japan, have a high incidence rate of MMD (three cases per 100 000 paediatric patients). Comparatively, the incidence rate of MMD is ten times lower in Europe and all other countries, like North America, the US, and the Middle East^11^. According to the most recent literature review, there are only around 20 cases of MMD reported in Saudi Arabia, and otherwise, the incidence rate in the Middle East is still not well known^12,13^.

Globally, there is a notable bimodal distribution in the age at which MMD first manifests itself. The bimodal peak comprises a substantial peak during the first ten years of life and a modest peak in the late 20s–30s. Notably, variations in the distribution of sexes by geography have been noted. While the sex ratio is 1:1 in China, the incidence of MMD in females has been observed to be higher than that in males in foreign countries, with the male-to-female ratio ranging from (1:1.8) to (1:2.2)^14^.

To the best of our knowledge, this case represents the first documented case of MMD in Palestine.

### Pathophysiology and risk factors

For pathophysiology, in MMD, alterations in blood vessels may be linked to a diminished response to inflammation or malfunctioning cellular repair mechanisms^15^. Elevated levels of angiogenesis-related factors that promote arterial growth and neovascularization, such as vascular endothelial growth factor (VEGF)^16^, endothelial colony-forming cells^17^, basic fibroblast growth factor (bFGF)^18^, transforming growth factor beta-1 (TGFB1)^19,20^, and many cytokines, have been correlated to these alterations^21^. All of the previous factors may contribute to the pathogenesis. Patients with Moyamoya may have high amounts of fibroblast growth factor in their cerebrospinal fluid^22^ and vascular intima, media, and smooth muscle^18^. Additionally, elevated levels of hepatocyte growth factor, a potent stimulator of angiogenesis, have been observed in the carotid arteries and cerebrospinal fluid of Moyamoya patients^23^. The intracranial portion of the internal carotid arteries, the proximal portion of the middle cerebral artery, and the anterior cerebral arteries are the most commonly affected vessels, with the sparing of the posterior arteries of the Circle of Willis in most cases^24^.

MMD-specific causes are still unexplained, but the fact that it has a higher prevalence among East Asians and reported familial cases raised suspicion for genetic underlying causes^14^. The RNF213 gene on chromosome 17 was identified as an important risk factor for MMD through multiple genomic studies done on East Asian descendants^25^. The incidence of MMD increases in patients who have sickle cell disease, Down’s syndrome, and neurofibromatosis type I^26^. Other specific human leucocyte antigen haplotypes on chromosomes 3, 6, and 8 have been described in the literature. Even though environmental factors are thought to precipitate the disease in potentially predisposed patients, as evidenced by reports of one affected sibling of an identical twin^24^, So, as mentioned above, our patient is part of a twin; the other sibling is completely healthy.

### Clinical presentation

Asymptomatic, ischaemic, or haemorrhagic presentations are all possible in MMD. Ischaemic events are considered the most common initial presentation, especially in children. Haemorrhagic presentation is significantly rare in the paediatric population; however, it occurs later in life with the disease’s progression. Loss of acquired developmental milestones is significant in paediatric patients with MMD. Headache of migraine-type vascular headache is common in MMD patients. MMD patients are also at increased risk of developing seizures^27^. Ischaemic events have been observed to be precipitated by different factors: hyperventilation, hypocarbic, hypoxic, hypotensive, or hyperthermic. Ischaemia occurs when the stenotic vessels are unable to compensate^28^.

### Diagnosis

The Moyamoya Disease Research Committee has been studying the diagnosis and treatment of MMD since it was established in Japan in 1974. From then on, the criteria have undergone four revisions to reflect changing concepts of disease and advancements in diagnostic imaging. The most recent revision was made in 2021 and focused on the primary diagnostic modality, highlighting radiological findings in cerebral angiography and MRA and concluding with the exclusion of other diseases that could be associated with the confusing name of Moyamoya syndrome, which can be distinguished from Moyamoya disease by its unilateral involvement of the brain and its secondary nature, such as radiation exposure. See Table 1 ^29^.

**Table 1:** Diagnostic criteria 2021 of Moyamoya disease

(A) Radiological findings	(1) Cerebral angiography: (1) Stenosis or occlusion in the arteries centred on the terminal portion of the intracranial internal carotid artery.(2) Moyamoya vessels (abnormal vascular networks) in the vicinity of the occlusive or stenotic lesions in the arterial phase. Note: Both bilateral and unilateral cases can be diagnosed as moyamoya disease. 2. MRI and MRA: (1) Stenosis or occlusion of the terminal portion of the intracranial internal carotid artery. (2) Decrease in the outer diameter of the terminal portion of the internal carotid artery and the horizontal portion of the middle cerebral artery bilaterally on heavy T2-weighted MRI. (3) Abnormal vascular networks in the basal ganglia and/or periventricular white matter on MRA.
(B) Differential diagnosis: Moyamoya disease is a disease of unknown aetiology, and similar cerebrovascular lesions associated with the following should be excluded as quasi Moyamoya disease or Moyamoya syndrome	(1) Autoimmune disease (systemic lupus erythematosus, antiphospholipid syndrome, polyarteritis nodosa, Sjögren syndrome, etc.), (2) Meningitis, (3) Brain tumours, (4) Down’s syndrome, (5) Neurofibromatosis type 1, (6) Cerebrovascular lesions after head irradiation. Note: Cases with hyperthyroidism can be diagnosed as moyamoya disease.

(Diagnostic Assessment Moyamoya disease) (1) and (2) of A-1 or (1) to (3) of A-2 are met and B is excluded.

MRA, magnetic resonance angiography.

Suzuki is a staging system that has been utilized to quantify the severity of Moyamoya angiographically by considering the emergence of Moyamoya vessels and the development of collaterals from the ECA. The initial stages^1–3^ include the appearance of steno-occlusive changes in the region of the ICA terminus and the subsequent development of ‘moyamoya vessels’, whereas in the 4th and 5th stages, the transdural and/or transcranial anastomoses from the ECA develop with the consequent disappearance of ‘moyamoya vessels’, followed by the ‘burnt out’ stage (stage 6), where the intracranial internal carotid and the collateral circulation from the external carotid disappear^10^.

It is worth mentioning that Suzuki staging doesn’t correlate with the clinical picture of the Moyamoya patients; different stages of Suzuki can present with a similar clinical picture as the complex pathology of Moyamoya is not fully understood, and the different compensating mechanisms are not fully considered in the Suzuki staging^30^.

### Treatment

Until now, MMD has been a non-curative disease. Medical management aims to prevent complications of the disease; antiplatelet agents are used to reduce the formation of thromboses. MMD patients are at higher risk for aneurysm formation in relation to the general population, even though endovascular therapy with stent placement has failed to treat the aneurysms of MMD patients^31,32^. The most effective treatment option for MMD is surgical revascularization, whether direct, indirect, or combination. This will help to restore cerebral blood flow to the affected areas, prevent further strokes, and improve neurological outcomes^27^.

In direct techniques, an external carotid artery-to-internal carotid artery (ECA-to-ICA) bypass is created by direct anastomosis between the distal branches of the ECA [superficial temporal artery (STA) and ophthalmic artery(OA)] and the distal branches of the ICA [M4 segment of the middle cerebral artery(MCA)]. Sometimes, the middle meningeal artery (MMA) can be used in cases of damaged STA. The success rate of ECA-to-ICA bypass ranges from 87 to 100%. This procedure immediately reestablishes blood flow after the anastomosis is created^33^.

In indirect techniques, a new anastomosis is created between internal and external circulations. Procedures depend on the angiogenesis that occurs in response to wound healing as a normal physiologic process mediated by different angiogenic signalling molecules such as fibroblast growth factor (FGF), (VEGF), neuropilin-1 (NRP-1), and angiopoietin-1 (Ang1). However, indirect bypass techniques take time for significant revascularization to occur^34^.

Encephalo-duro-arterios-ynangiosis (EDAS) is where the STA is moved directly to the surface of the cerebrum, and eventually, anastomosis between the STA and MCA will form and so increase blood flow to the hypo-perfused areas of the brain^35^. Omental-cranial transposition involves omental flap suturing into resected Dural margins, angiogenesis formation, and anastomosis between the omentum and MCA distal branches^36^. Encephalo-myo-synangiosis (EMS) is where the temporalis muscle is sutured into resected dura mater^37^. Encephaloduroarteriomyosynangiosis (EDAMS) is a combination of indirect EMS and EDAS procedures. A vascular-rich muscle graft and vessel movement are done simultaneously. Any distal ECA branch is placed onto the arachnoid surface close to distal M4 MCA. On both sides of the ECA, the grafts are laid, and together they will facilitate angiogenesis^27^.

Studies show that direct methods give immediate results; however, indirect methods have a more favourable clinical course, especially if done at the early stages of the disease, and a lower incidence of future strokes^38^.

## Conclusions

This case report highlights the clinical presentation, diagnostic challenges, and treatment considerations for MMD. The patient presented with recurrent episodes of convulsions, leading to a thorough diagnostic evaluation that included an electroencephalogram, brain CT, MRI, MRA, MRV, and cerebral catheterization. The timely identification of MMD is crucial for implementing surgical revascularization as soon as possible to prevent complications.

## Ethical approval

Not applicable.

## Consent

Written informed consent was obtained from the patient’s family for reporting this case and its associated images. The consent is available for review on request.

## Source of funding

Not applicable.

## Authors contribution

All authors fulfil the authorship criteria because of their substantial contributions to the conception, design, analysis, and interpretation of the data. H.M.N.H. wrote the introduction, participated in writing the discussion, reviewed the article, and collected the data. R.B.A. participated in discussion writing and wrote the conclusion. A.A. participated in discussion writing and abstract writing. A.A.A.D. wrote the case presentation, participated in data collection, and examined the patient. Y.M.N.H. participated in writing the discussion and introduction, corrected grammars. M.A. reviewed the article, prepared the final submission, and submit the article. S.I.B. The consultant who treated the patient, reviewed the article.

## Conflicts of interest disclosure

The authors declare that they have no competing interests.

## Research registration unique identifying number (UIN)

Not applicable.

## Guarantor

Yousef Mahmoud Nimer Habes.

## Data availability statement

Not applicable.

## Provenance and peer review

Not commissioned, externally peer-reviewed.
